# Supplementary material for: The vacuolar iron transporter mediates iron detoxification in Toxoplasma gondii
Source: Nat Commun. 2023 Jun 20;14:3659. doi: 10.1038/s41467-023-39436-y (PMC10281983; doi:10.1038/s41467-023-39436-y)
Supplement: Supplementary file 1 — Supplementary information [file 41467_2023_39436_MOESM1_ESM.pdf]

```
                1
Tb927.3.800     ---------- ---------- -----MSESE KIDVSGARNY KS-----ISA ARXKAFETGD
PF3D7_1223700   ---------- ---------- ---------- -------MVS KK-----TIE ARXKAYYNED
PBANKA_1438600  ---------- ---------- ---------- -------MGK QK-----IID ARXKAYYEGD
ETH_00007450    ---------- ---------- --MAQLMGDE ERRDVYSATT NA-----LQT RFXYAMYIRA
TgVit           ---------- -------MPA SGAGYAGGSI VPDGRTLVNC RD-----LHK ARXDAYQLRD
NCLIV_039240    ---------- -------MPA GGAGHAGGNI VPDGRTLVNC RD-----LNK ARXDAYQLRD
ScCCC1          MSIVALKNAV VTLIQKAKGS GGTSELGGSE STPLLRGSNS NSSRHDNLSS SSSDIIYGRX
AtVit1          ---------- ---------- ---------- ---------- -------XMS SEEDKITRIS
EgVIT1          ---------- ---------- ---------- ---------- ------XMAD GANDGGNPGA

                                                                     Asp43 Gly44
               61
Tb927.3.800     IEMSRMEHQK HI--YKEV-- -----H---- ----NPSASD YVKSVVFGGL DGXIITSFTV
PF3D7_1223700   VVLSKEAHDF ----YHNL-- DKHGEN---- ----HNLDKD NLKTIIFGSL DGXIITIFAI
PBANKA_1438600  IEKSKEIHSH ----YHNL-- DKHAEH---- ----HSLDKD HLKTIIFGSL DGXIITIFAI
ETH_00007450    AHQHNHHHSQ NPGDYGELSG VHTEAH---- ----KKTSSD YLKAIVFGGL DGXIVTIFAI
TgVit           IEATRAAHSL DL--YRELTG DHKENH---- ----TNTSSD YVKAVVFGGL DGXIVTIFAI
NCLIV_039240    IEATRAAHSL DL--YRELAG DHKESH---- ----TNTSSD YVKAVVFGGL DGXIVTIFAI
ScCCC1          NSAQDLENSP MSVGKDNRNG DNGSDNEKAN LGFFQSVDPR VISDLIIGLS DGXLTVPFAL
AtVit1          IEPEKQTLL- ---------- DHHTEK---- ----HFTAGE IVRDIIIGVS DGXLTVPFAL
EgVIT1          EEQQRL---- --------L-- DQHKEA---- ----HFTAGE IVRDIIIGVS DGXLTVPFAL

                              Gly69 Glu72 Gly76 Met80                 Glu102 Glu105
               121
Tb927.3.800     VSAAVGSNSS VASVLIFGFS NVIADGFAMG FGEYVSGEAE RDNALSERXR REEWEVENAF
PF3D7_1223700   VSGCVGAKIT PTQVIIIGIG NLFANAISMG FSEYTSSTAQ RDFMLAEKXK REEWEIENCP
PBANKA_1438600  VSGCVGANIT PAQVIIIGVG NLFANAISMG FSEYTSSTAQ IDFMLAERXQ REEWEIENCP
ETH_00007450    VAGCVGANLH PSKVVIIGIG NLLADAISMG FGEFVSSAAE DDFVKSERXD REEWEIENCP
TgVit           VAGCVGADLS CSQVLMVGLG NLLADAISMG FGEYVSAAAE KDFVEAEKXQ REEWEVENCP
NCLIV_039240    VAGCVGADLS CSQVLMVGLG NLLADAISMG FGEYVSAAAE KDFVEAEKXQ REEWEVENCP
ScCCC1          TAGLSSLG-D AKLVITGGFA ELISGAISMG LGGYLGAKSE SDYYHAEVXK KEKRKFYDNS
AtVit1          AAGLSGANAS SSIVLTAGIA EVAAGAISMG LGGYLAAKSE EDHYAREMXK REQEEIVAVP
EgVIT1          AAGLSGANAS SSIVLTAGIA EVAAGAISMG LGGYLAAKSE ADNYARELXK REQEEIIRVP

                Glu113 Glu116                              Met149 Glu153
               181
Tb927.3.800     DMEVDEMVQI YE--MKGLSH EDATTIVNII SKDPKLFVDF MMTEELGIXI IDTEDTHGPK
PF3D7_1223700   SEEKQEMIDI YMN-KYKFDS EDARNLVEIT FRNKNFFLEH MMSEELGLXI VTNEDKNECL
PBANKA_1438600  TEEKQEMIDI YIN-KYKFDS KDAKNLVEIT FRNKHFFLEH MMSEELGLXI LTNEDKSEAF
ETH_00007450    DEEKQEMIEI YRD-RYGFTE EDADSLVNIT FKYREFFVRH MMVEELGLXM -ATEGPS-PL
TgVit           EEEKREMVEI YTE-KYGFSR ADAQSMVDIT FKYKKFFVQH MMVEELGLXM YGFDEPT-PI
NCLIV_039240    EEEKREMVEI YTE-KYGFSR ADAQSMVDIT FKYKKFFVQH MMVEELGLXM YGFDEPT-PI
ScCCC1          NLINREIEDI LLEINPNFSD ETIVSFIKDL QRTPELMVDF IIRYGRGLX- -DEPAENREL
AtVit1          ETEAAEVAEI LA--QYGIEP HEYSPVVNAL RKNPQAWLDF MMRFELGLX- -EKPDPKRAL
Eg_VIT          DTEAAEVAEI LA--RYGIEP HEYGPVVNAL RKKPQAWLDF MMKFELGLX- -EKPDPKRAL

                                  Asp175
               241
Tb927.3.800     KQGLVMFLSF MFFGAVPLLA Y-----LPGK GKGIDGVFAL XSCFLATCAL IVLGMLRGYL
PF3D7_1223700   KKGIIMFLSF AVFGIIPLSA YVAYTVFFG- YTDYTTSFLV XVFISTLTTL FILGLFKSQF
PBANKA_1438600  KKGILMFLSF CFFGMIPLFS YVLYNLFFS- AENYTSSFAV XVFISTLITL FILGLFKSQF
ETH_00007450    RRGAVMFASF SIFGLLPLAG FVAWLTLSGT STDGHLAFAM XACVVSGIAL FILGFFKGRF
TgVit           KRGLVMFTAF CFFGLLPLAG FIGWVAAFGL GAEADMAFLM XACVVSIMTL FILGFSKGKF
NCLIV_039240    KRGLVMFTAF CFFGLLPLAG FIGWVAAFGL GAEADMAFLM XACVVSILTL FVLGFSKGKF
ScCCC1          ISAVTIGGGY LLGGLVPLVP Y-----FF-- VSDVGTGLIY XSIIVMVVTL FWFGYVKTKL
AtVit1          QSAFTIAIAY VLGGFIPLLP Y-----ML-- IPHAMDAVVA XSVVITLFAL FIFGYAKGHF
EgVIT1          QSAFTIAIAY VLGGLVPLIP Y-----MF-- IPVARKAVVA XSVILTLMAL LIFGYAKGYF

               301
Tb927.3.800     ------SGVS MLRSAALMVF NGVVSGLFSF TXVGSLVEHA LRSSIEVX-
PF3D7_1223700   ------TNQK PITCALYMVL NGMIAGMVPF LXLGVVLKNN ISEX-----
PBANKA_1438600  ------TTQK PIVCALSMVL NGSIAGMLPF LXFGVLLKTN SGDX-----
ETH_00007450    ------VNQS SLKSGLLMII NGTCAGTVAY TXVGAALEGV VAGTLX---
TgVit           ------VGQN PTKSACLMAM NGGCAGTVAY GXVGSLLQLV VGANLTAAX
NCLIV_039240    ------VGQN PTKSACLMAL NGSCAGTVAY GXVGSLLQLA VGANMSSGX
ScCCC1          SMGSGSSTSK KVTEGVEMVV VGGVAAGAAW FXFVKLLGX- ---------
AtVit1          ------TGSK PLRSAFETAF IGAIASAAAF CXLAKVVQHX ---------
EgVIT1          ------TDNK PFKSALQTAL IGAIASAAAF GXMAKAVQSX ---------
```

Figure S1. Full alignment of *T. gondii* VIT with VIT sequences from selected eukaryotes. Blue boxes represent transmembrane domains, purple indicates Asp43 and Met80 which are conserved in all VIT members and important for iron binding. Light purple indicates residues important in EgVIT iron binding which are not conserved in apicomplexan sequences. Red indicates conserved residues in the MBD important for Zn coordination. Orange residues indicate conserved 'kink-inducing' residues, important for metal binding the cytoplasmic pocket. All residue numbers from EgVIT.

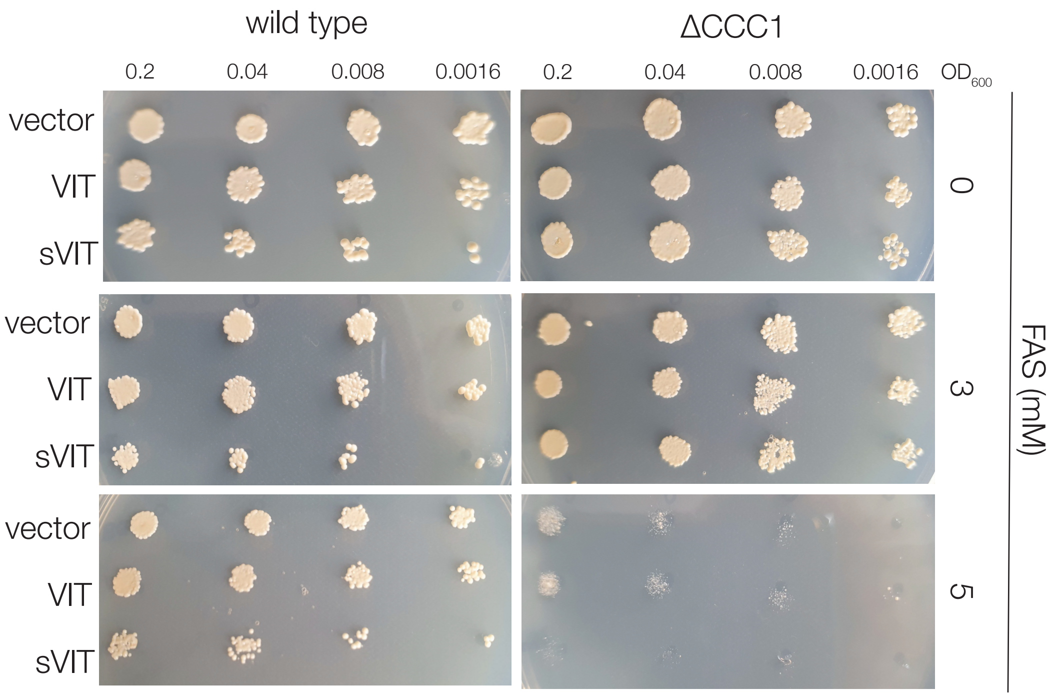

Figure S2. Complementation of yeast with TgVIT. Wild type or ΔCCC1 yeast were transfected with the empty vector or a vector expressing VIT or $sVIT_{63\text{-}313}$ (an N-terminal truncation) and plated at the indicated OD_{600} dilution . While the ΔCCC1 strain behaved as expected and was unable to grow on plates containing high iron, expression of VIT or $sVIT_{63\text{-}313}$ did not complement the phenotype.

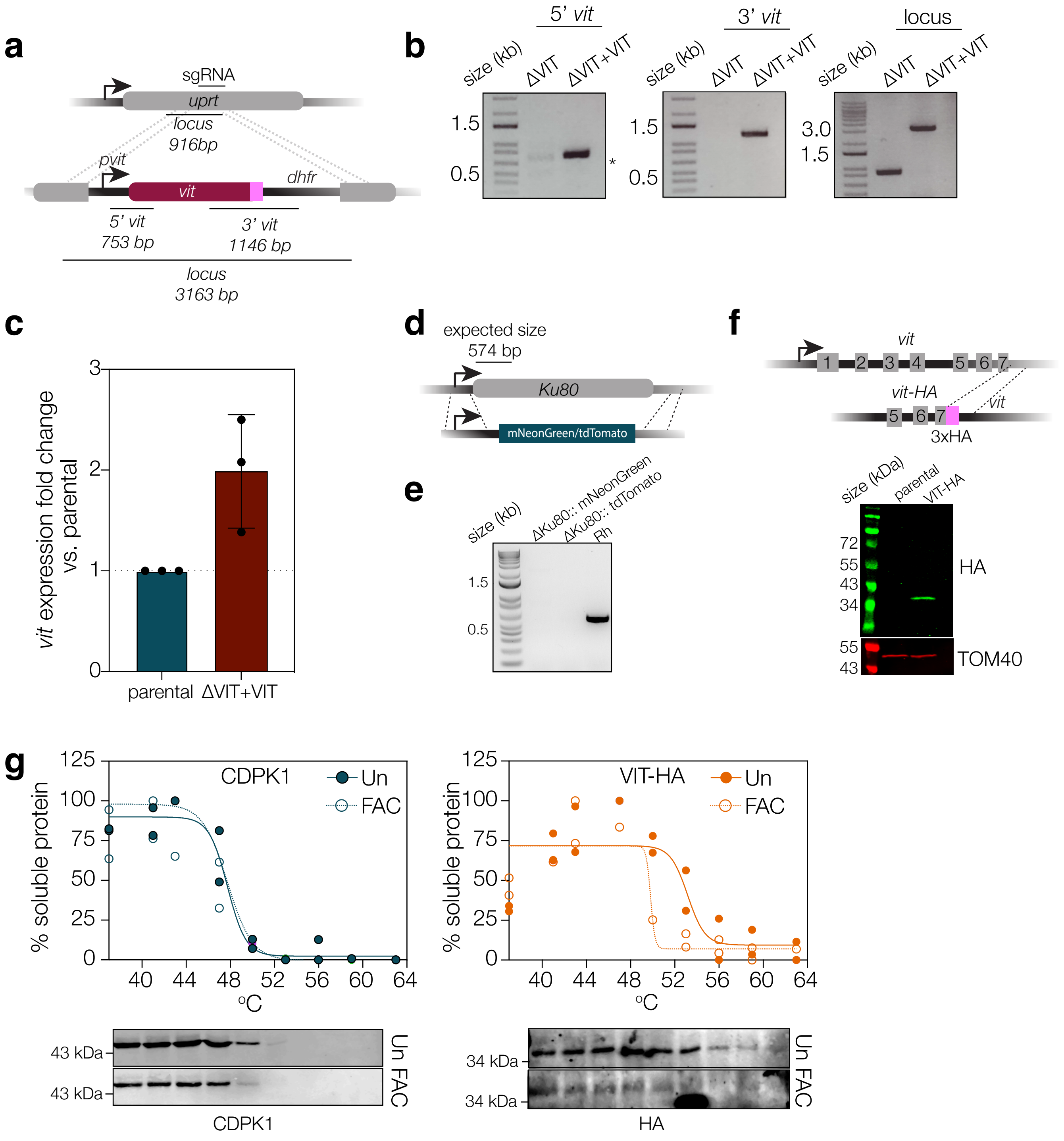

Figure S3. Complementation of ΔVIT parasite line and creation of new ΔKu80 lines expressing mNeonGreen or tdTomato a. Schematic indicating the strategy used to replace the uprt gene with the pvit-vit-3'dhfr cassette. **b.** PCR confirmation of the presence of the vit complementation cassette and the disruption of the endogenous locus, expected sizes indicated in (**a**). Non-specific band marked with asterisk. **c.** qPCR of *vit* in the complemented line shows approximately double the expression compared to parental line. Results are mean ± SD from three independent experiments. Schematic indicating the strategy for replacement of *ku80* gene by mNeonGreen or tdTomato cassettes. **e.** PCR of region indicated in (**d**), confirming the loss of the endogenous gene. **f.** VIT was endogenously tagged at the C-terminal with 3xHA tags. A single band at the expected size (35 kDa) was obtained. **g.** Lysates of VIT-HA parasites treated or untreated for 1 h with FAC were subjected to a range of temperatures as indicated and western blots using anti-CDPK1 and anti-HA performed. Band density plotted, normalised to 37 °C samples, points from two independent experiments.

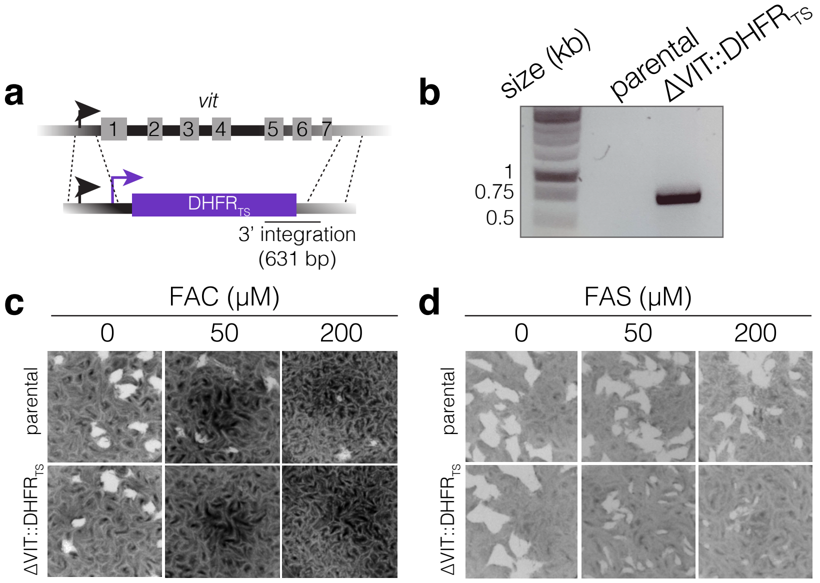

Figure S4. Construction of ΔVIT::DHFRTS parasite line. **a**. Schematic showing how *vit* was replaced with the DHFR cassette. **b.** PCR showing the 5' integration of the DHFR cassette. Plaque assays showing increased sensitivity of the ΔVIT::DHFR$_{TS}$ line to excess ferric ammonium citrate (FAC) **(c)** and ferrous ammonium sulphate (FAS) **(d)** at the indicated concentration compared to the parental parasite line. Representative plaque assays from three independent replicates.

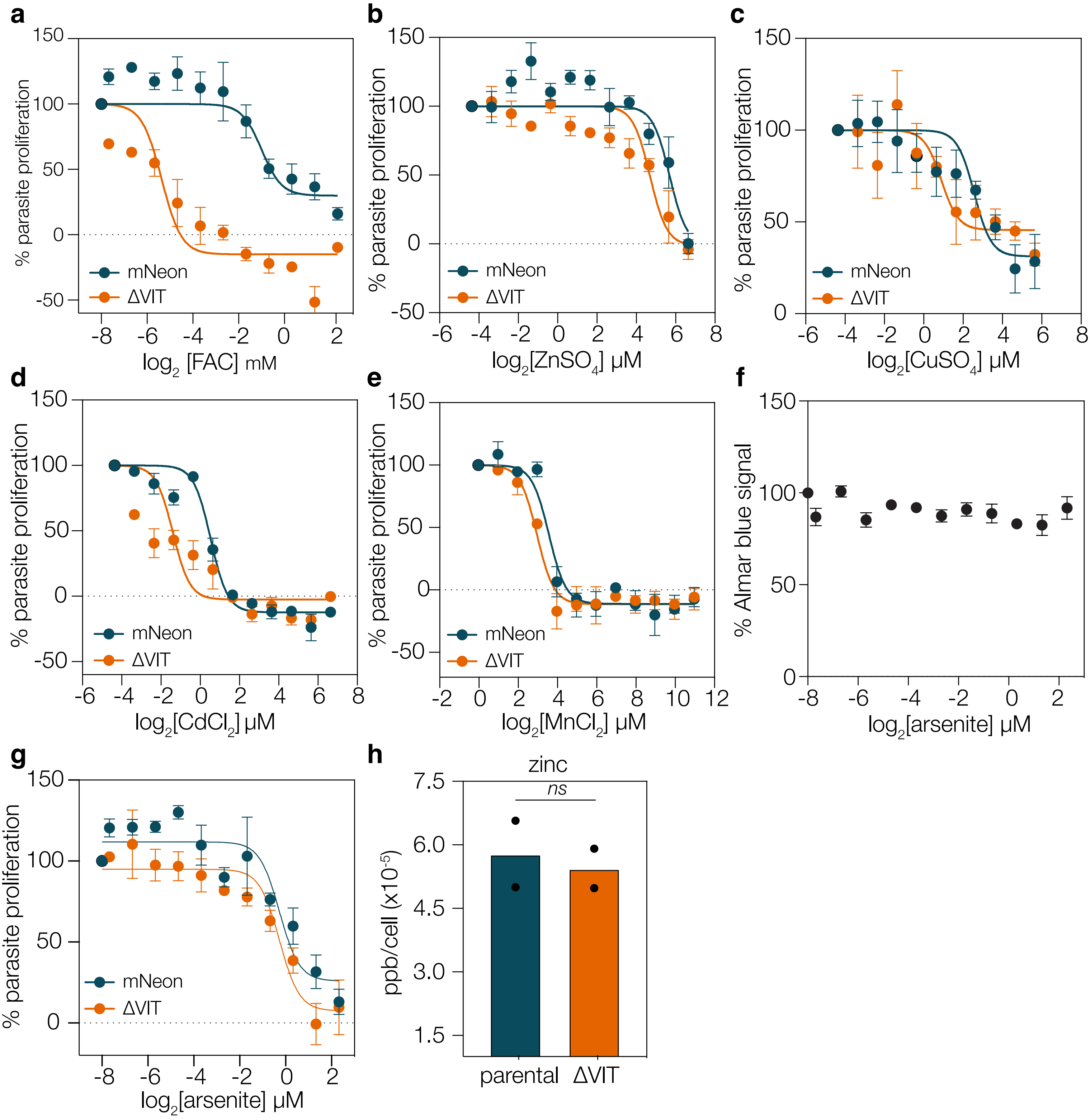

Figure S5. Dose response curves of mNeon and ΔVIT treated with excess metals. As described above, parasites were treated with the indicated concentration of FAC (**a**) $ZnSO_4$ (**b**), $CuSO_4$(**c**), $CdCl_2$(**d**), or $MnCl_2$ (**e**). All experiments are the mean of six (FAC) four ($ZnSO_4$) or three ($CuSO_4$, $CdCl_2$, and $MnCl_2$) independent experiments performed in triplicate, ± SEM. **f.** Survival of uninfected HFF cells at the range of sodium arsenite concentrations used above. No change in fluorescence was seen at the concentrations used. Results are the mean n = 3 independent bioloical replicates, ± SEM. **g.** Treatment of mNeon and ΔVIT parasites with sodium arsenite, a ROS generator, showed no difference in sensitivity between the two lines. Points are the mean of n = 3 independent bioloical replicates, ± SEM. **h.** ICP-MS quantifying zinc (^{66}Zn) from parental and ΔVIT parasites. Each point represents an independent biological replicate, performed in technical duplicate. Bars at mean.

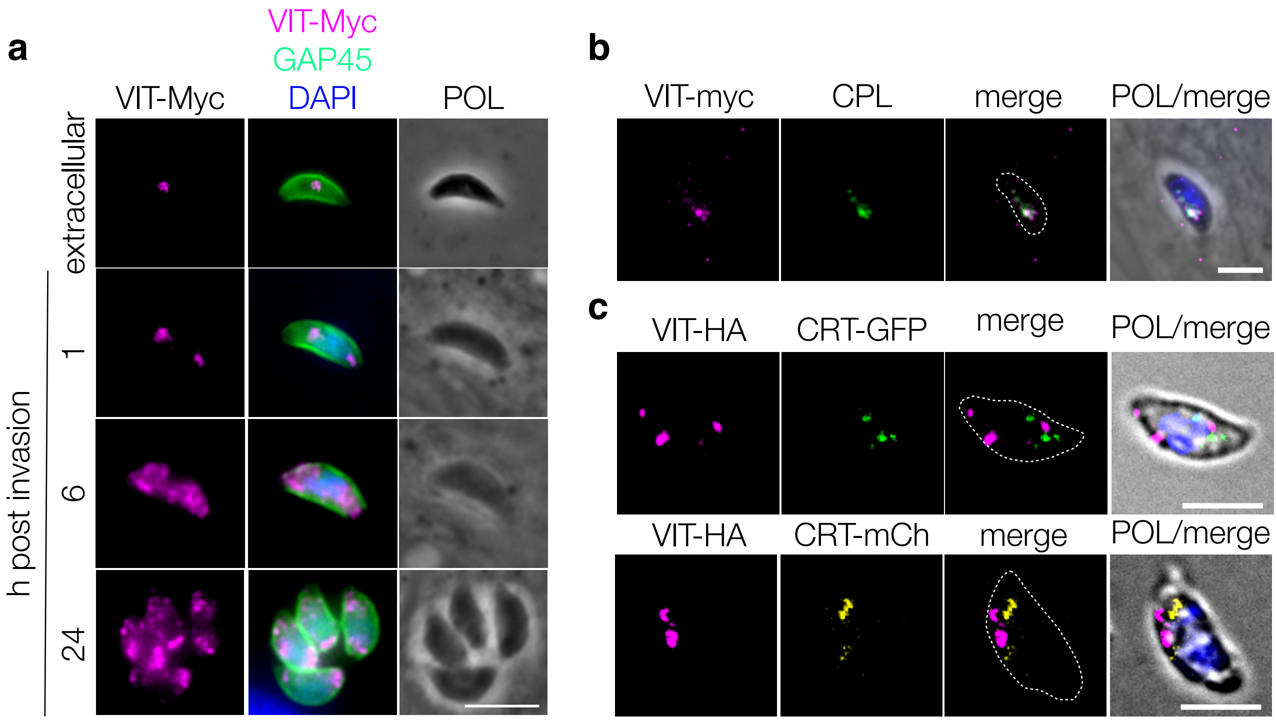

Figure S6. VIT-myc has a dynamic localisation. a. VIT-myc demonstrates a dynamic localisation throughout the cell cycle and fragments between 1 and 6 h post invasion. Cell periphery indicated by GAP45. Representative of three independent experiments. **b.** VIT-myc colocalizes with CPL in extracellular parasites. Representative of two independent experiments. **c.** VIT-HA does not colocalize with transiently overexpressed CRT-GFP or CRT-mCherry in extracellular parasites. Representative of one experiment. Scale bar 5 µm.

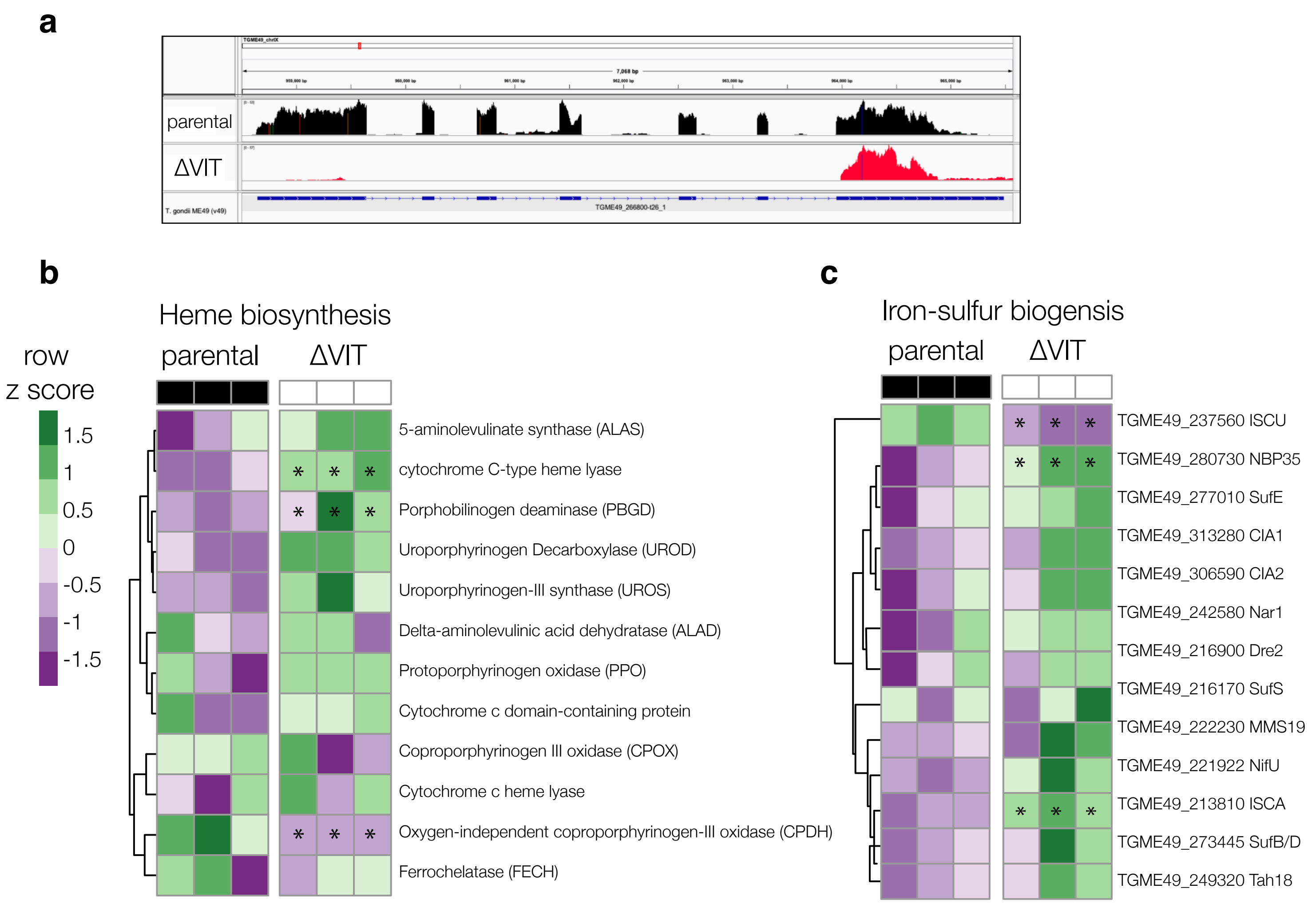

Figure S7. Heatmaps of genes from major iron pathways from parental and ΔVIT parasites a. Mapping of all reads from the parental and ΔVIT strains to the vit gene. As the 5' UTR of *vit* still exists, some reads map to this region, but no reads map to the coding regions**. b**. Genes involved in the heme biosynthesis pathway, read counts normalised across rows. * indicates adj. p value of < 0.05 between parental and ΔVIT strain. **c.** Genes involved in Fe-S biogenesis read counts, normalised across rows. * indicates adj. *p* value from Wald test with Benjamini and Hochberg correction of < 0.05 between parental and ΔVIT strain.

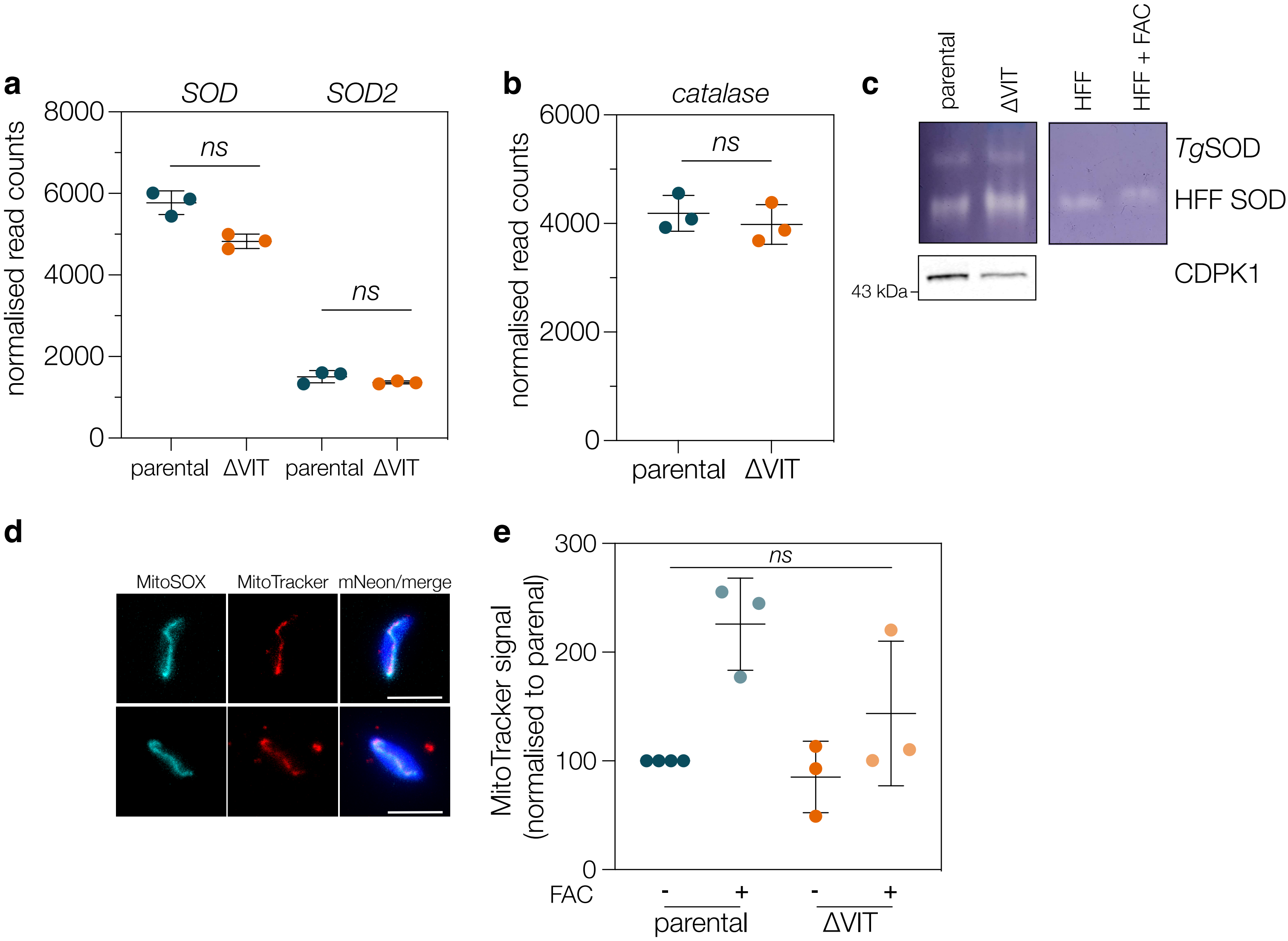

Figure S8. ΔVIT parasites do not alter transcription of oxidative stress genes a. Normalised read counts for *SOD* (TGME49_316310) and *SOD2* (TGME49_316330) from RNAseq. Each point represents an independent experiment, performed in duplicate. Line at mean ± SD. *ns*-non significant from Wald test with Benjamini and Hochberg correction. **b.** Normalised read counts for catalase (TGME49_232250). Each point represents an independent experiment, performed in duplicate. Line at mean ± SD. *p* value from Wald test with Benjamini and Hochberg correction . **c**. In gel activity assay for SOD, enzyme activity results in cleared areas on native gel. Host (HFF) and parasite enzymes indicated, CDPK used as a parasite loading control. No obvious differences were seen between the parental and ΔVIT parasite lines. Results representative of two independent experiments. **d.** Live mNeon parasites were co-stained with MitoSOX Red (cyan) and Mitotracker DeepRed (red) and imaged. MitoSOX and MitoTracker signals co-localised in the mitochondrion of the parasites. Scale bar 5 µm. **e**. MitoTracker signal was measured by flow cytometry and normalised to the mNeon untreated line. Although FAC treatment led to generally increased signal, no significant differences were seen (Kruskal-Wallis test, Dunns multiple comparison correction). Each point represents an independent experiment, $n = 3$, line at mean ± SD.

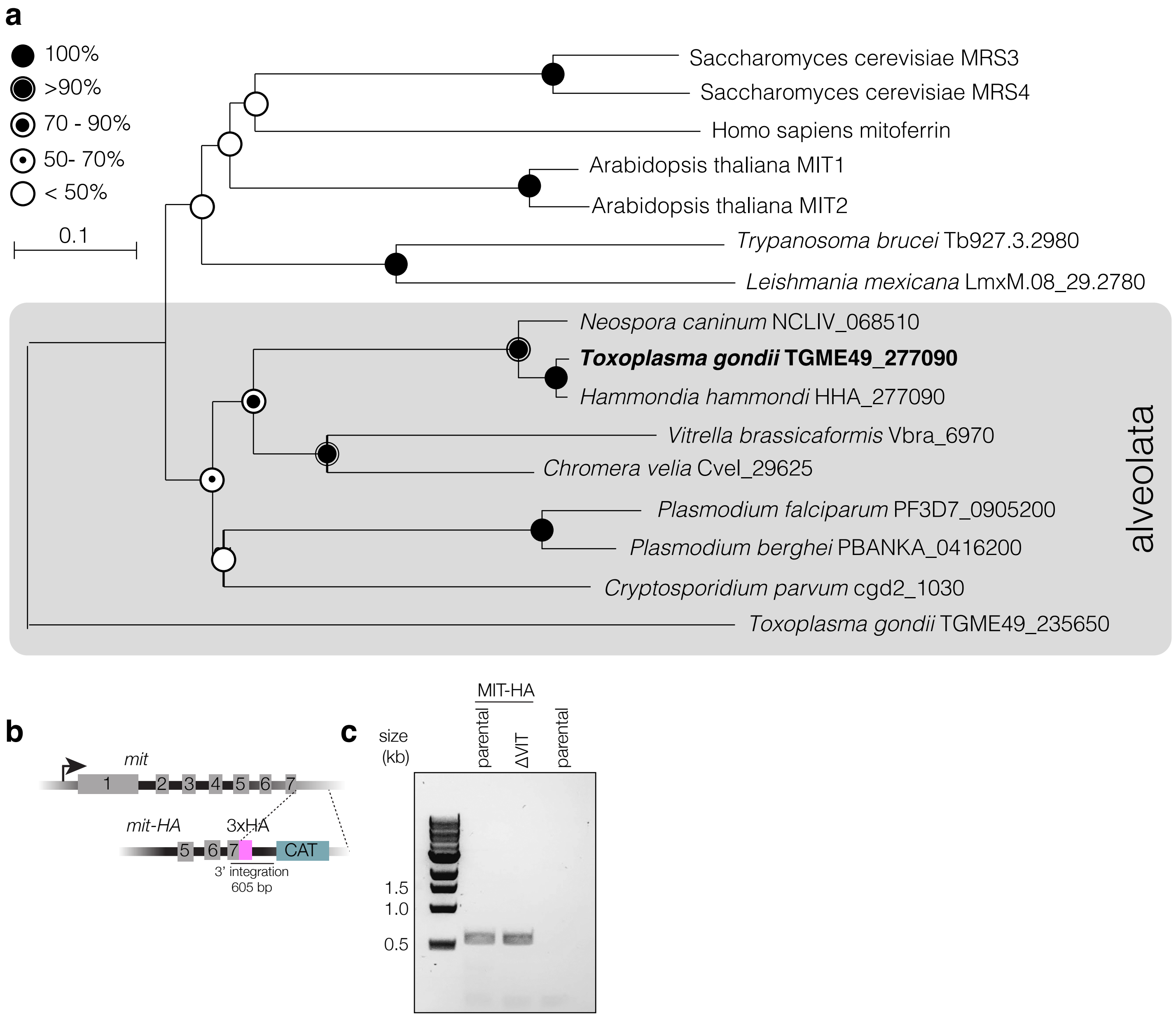

Figure S9. Identification and characterisation of *T. gondii* mitochondrial iron transporter (MIT). a. Phylogenetic tree of MIT sequences from a variety of species. TGME49_235650 represents an unrelated mitochondrial solute carrier. Bootstraps determined from 100 simulations. Scale indicates substitutions per site. **b**. Diagram demonstrating C-terminal HA endogenous tagging strategy. **c.** PCR confirming the integration of HA into MIT locus in the parental (Δku80) and ΔVIT parasite lines.

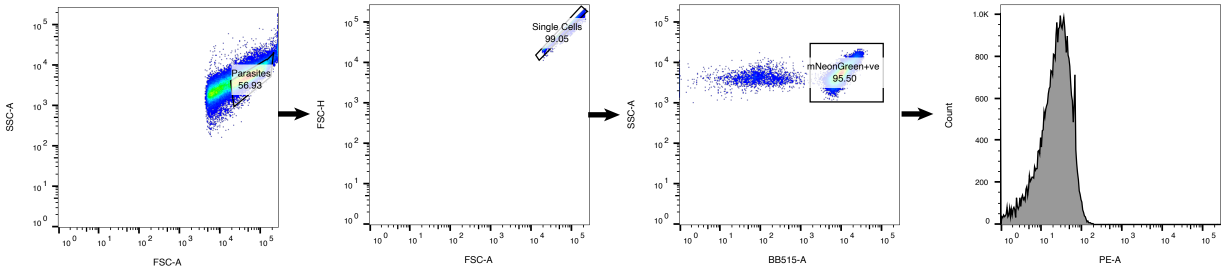

Figure S10. Example gating strategy Δku80::mNeonGreen parasites were stained with MitoSOX as described. Gates were drawn as indicated to isolate parasites, remove doublets and select only mNeonGreen parasites to calculate geometric MFI.
