# Supplementary material for: The vacuolar iron transporter mediates iron detoxification in Toxoplasma gondii
Source: Nat Commun. 2023 Jun 20;14:3659. doi: 10.1038/s41467-023-39436-y (PMC10281983; doi:10.1038/s41467-023-39436-y)
Supplement: Supplementary file 3 — Description of Additional Supplementary Files [file 41467_2023_39436_MOESM3_ESM.docx]

**Description of additional supplementary files**

Title: Supplementary Dataset 1

Description: Processed RNAseq data for parental and ΔVIT parasites

Title: Supplementary Figures

Description: Supplementary figures S1-S10
